# Correlation of Ambient Temperature With Feedlot Cattle Morbidity and Mortality in the Texas Panhandle

**DOI:** 10.3389/fvets.2020.00413

**Published:** 2020-08-04

**Authors:** Paul R. Broadway, Steven A. Mauget, Nicole C. Burdick Sanchez, Jeffery A. Carroll

**Affiliations:** ^1^USDA, ARS Livestock Issues Research Unit, Lubbock, TX, United States; ^2^United States Department of Agriculture (USDA), Agricultural Research Service (ARS) Wind Erosion and Water Conservation Research Unit, Lubbock, TX, United States

**Keywords:** cattle, feedlot, health, morbidity, mortality, temperature

## Abstract

Anecdotal data would suggest that weather patterns influence beef cattle health in feedyards, and cattle producers often associate the seasonality of some illnesses with changes in environmental temperatures. However, to our knowledge, there is little information from large-scale feeding operations and precision weather stations that establishes a link or lack thereof between weather patterns and cattle health. Additionally, we are unaware of any studies correlating other weather parameters with animal health data. Therefore, the objective of this study was to test for associations between monthly temperature variation and animal morbidity/mortality in feedlots in the Texas Panhandle. Weather data was collected from a Texas Tech University Mesonet weather station in close proximity to 19 beef cattle feedyards in the Texas Panhandle. Additionally, near real-time morbidity and mortality data was collected from those yards from 2015 to 2018. These data document a seasonal pattern relative to cattle morbidity and mortality with most health events occurring from November to January. This pattern is differentiated when comparing morbidity and mortality by listed causation (e.g., respiratory, digestive, other), and the majority of deaths over the entire time course were attributed to respiratory disease. Most cattle morbidity was documented in the winter months, most of which were classified as respiratory disorders. Additionally, an increase in health events was observed as the population of the feedyard increased. However, the overall effects of ambient temperature on cattle health were minimal and the two may not be causally linked. The initial overview of the relationships documented in this manuscript may warrant further stratification and exploration.

## Introduction

Extreme weather events such as heat waves, blizzards, ice storms, hurricanes, tornadoes, and floods pose a risk to feedlot cattle health, productivity, and well-being. For example, according to the National Weather Service, a March 1957 blizzard resulted in the death of ~20% of all cattle in the Texas and Oklahoma Panhandle (NOAA, NWS). More recently, a 2016 winter storm was responsible for the deaths of over 30,000 head in Texas and New Mexico (1). Hurricane Harvey in 2017 resulted in thousands of head reported lost or dead in addition to the millions in dollars of equipment and feed losses (2).

Not only do these events pose a direct physical threat to cattle mortality, but also they may lead to secondary long- and short-term effects on cattle health and well-being (3). Economic losses, stemming not only directly from morbidity and mortality but also due to performance losses, may accompany weather events. Peel (4) reported that winter weather patterns may also disrupt feedlot placements and influence cattle markets. Other research reported that feed efficiency and animal performance may be negatively impacted due to climate and weather patterns (5). For example, a 2003 report suggested that heat stress could be responsible for over $2 billion dollars in losses annually across different sectors of livestock production (6). Additionally, Hubbard et al. (7) reported that prolonged and sustained high-temperature-humidity indices (THI) can result in increased feedlot cattle mortality.

Animal caretakers and veterinarians recognize weather patterns as an important factor contributing to feedlot cattle health. In fact, a survey of feedlot veterinarians reported weather as a concern or risk factor contributing to animal health (8). Anecdotally, producers often refer to Fall as a time of year in which morbidity is most prevalent in their operations, and they often attribute these health problems to extreme fluctuations in temperature throughout the day. There is also data to support claims that air temperature can influence cattle health (9, 10). Specifically, Cusack et al. (11) asserted that daily weather fluctuations may impact bovine respiratory disease (BRD) incidence. Data from Ribble et al. (12) further confirm seasonality of illness, reporting November feedlot placement as a risk factor for BRD when compared to other months.

Overall, literature correlating weather patterns to real-time morbidity and mortality is sparse and much of the previous literature does not cover a wide time frame or sample population. Therefore, the following data retrospectively characterize weather patterns for 4 years and its relationship to the corresponding incidence of morbidity and mortality in feedlot cattle in the Texas Panhandle using precision temperature-monitoring systems in close proximity to beef cattle feedlots.

## Materials and Methods

Ethical approval was not required according to national/local legislation because the described study did not use live animals. Rather, this manuscript describes a retrospective analysis of cattle data acquired from a database of U.S. feedyard data.

### Feedyard and Meteorological Data

**Figure 1:**
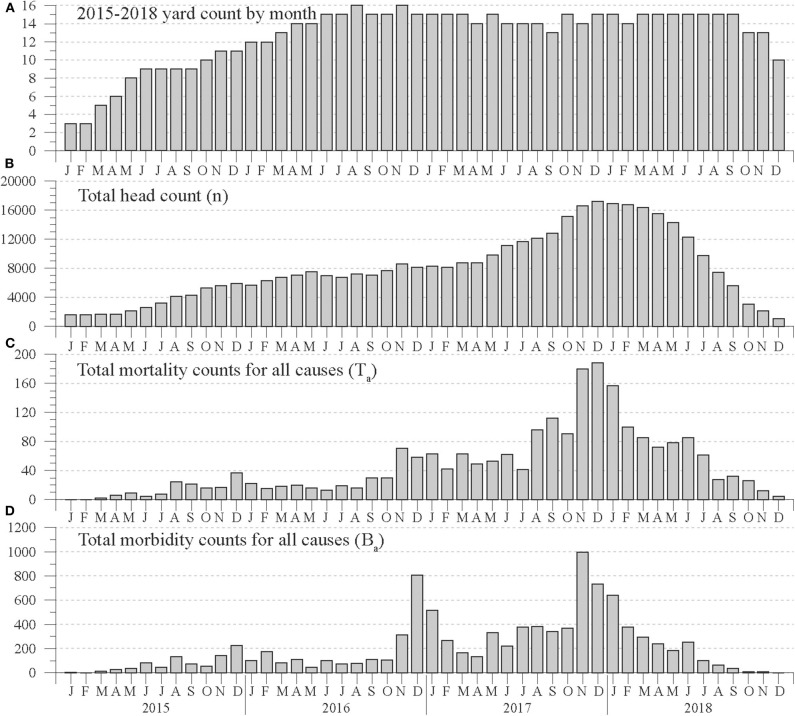
Monthly counts of **(A)** feedyards reporting population, mortality, and morbidity data, **(B)** estimated total head on feed, **(C)** total mortality, and **(D)** total morbidity counts for cattle within a 96.6-km radius of Hereford, TX, in the Texas Panhandle from January 2015 to December 2018.

In order to obtain real-time (daily) morbidity and mortality data and associated population and demographic data from feedlots, data were acquired from Elanco Knowledge Solutions (EKS) Benchmark Performance Program (Elanco Knowledge Solutions, Lenexa, KS, 2019). This database compiles real-time data acquisition from 254 beef cattle feedyards within the United States. For this analysis, data were only collected from feedlots in the Texas Panhandle. Specifically, beef cattle feedyards were included in the sample population within a 96.6-km radius of Hereford, TX. Daily data was collected from the date range: January 1, 2015, to December 31, 2018, based on the date of closeout for a feedlot defined lot. These feedlots were selected to prevent no more than 40% of the cattle on any given date to be owned by a single feedlot. Feedlot names, locations, and lot numbers were coded using a random-number generator as to blind data analysts and maintain anonymity using the RAND and RANK functions of Excel. Data were also sorted to remove any lots within a feedlot which took >30 days to fully place or fill. Data included both steers and heifers but were limited to only include beef-type cattle and exclude calf-feds and dairy beef. Data from lots fed > 400 days were excluded, and cattle placed in the lot were required to have an incoming weight <1,000 lbs. (453.59 kg). During the 48-month period, the number of yards contributing to morbidity and mortality counts during any month varied from 3 to 16 (Figure 1A). The monthly population, morbidity, and mortality counts calculated here were derived from daily head counts for lots in those yards in a given month. The total number of lots in a month's available data were generally proportional to the monthly total head count (Figure 1B) and varied from 24 in December 2018 to 219 in January 2018.

Concurrent to feedlot data, weather data was derived from the West Texas Mesonet [WTM; (13)] weather station at Hereford, TX. Daily minimum (TMIN) and maximum (TMAX) temperatures were derived from archived 5-min WTM Hereford temperature records. Given the possible association of cattle health with extreme daily shifts in temperature, daily temperature range (DTR) values (i.e., maximum minus minimum daily temperatures) were also derived from the 5-min Hereford temperatures. The resulting daily records were then averaged into monthly TMIN, TMAX, and DTR values for each month of January 2015 to December 2018.

For purposes of these analyses, morbidity was defined as a first hospitalization or treatment event in an attempt to better attribute weather patterns to initial illness and not confound morbidity as an additional treatment in response to a possible previously acquired illness or treatment event. Mortality was simply defined as a recorded death. Attribution of morbidity and mortality reports is assumed accurate as having entered into the real-time database and does not require clinical and/or pathological burden of proof which allows for possible misdiagnoses within the dataset. Furthermore, morbidity and mortality were stratified by causation. Aside from the aforementioned sorting and selection criteria, no other stratification of weather and/or morbidity and mortality data was performed, and all data were collapsed into similar time categories (e.g., month within year) prior to analysis.

### Statistical Methods

Monthly population counts for each lot in a feedyard were estimated as the average of the lot's daily head-in-pen counts during the month. Monthly total population, or total head count, was considered the sum of the average head counts of all lots in all feedyards during the month. Monthly death counts for all causes in a lot are the sum of deaths reported daily during the month due to all causes for the lot. In addition, daily death counts classified as respiratory, digestive, and other causes for each lot were summed into monthly counts for those causes. The feedyards' total monthly death counts for all (a), respiratory (r), digestive (d), and other (o) causes were tallied as the sum across all lots in all feedyards.

Because the number of feedyards reporting data from January 2015 to December 2018 varied widely, monthly total population counts varied proportionally (Figure 1B). As a result, mortality and morbidity counts were normalized to monthly percentages based on the same month's total population count. Thus, a month's mortality percentage (%_t_) was calculated as

(1a) $${\%}_{t}=100.0*\text{t}=\;100.0*\frac{\text{T}}{\text{n}},$$

where T is a monthly mortality count and n is the month's total population count. Similarly, morbidity percentages (%_b_) were calculated as

(1b) $${\%}_{b}=100.0*\text{b}=\;100.0*\frac{\text{B}}{\text{n}},$$

where B is a monthly morbidity count. For the three reported causes, monthly mortality (%_tr_,%_td_,%_to_) and morbidity (%_br_,%_bd_,%_bo_) percentages were calculated based on the t (t_r_,t_d_,t_o_) and b (b_r_,b_d_,b_o_) ratios of the corresponding mortality (T_r_,T_d_,T_o_) and morbidity counts (B_r_,B_d_, B_o_). The significance of differences between ratios was determined by comparing uncertainty intervals based on the standard deviation of a large sample of proportions, e.g.,

(2) $$\begin{array}{l} \sigma_{t}=\;\sqrt{\frac{t*\left(1-t\right)}{n}}, \end{array}$$

and conducting two-proportion Z-tests. Thus, for example, to compare two mortality ratios t_1_ and t_2_,

(3a) $$\begin{array}{l} Z\left(t_{1},\;t_{2}\right)=\;\frac{t_{1-\;}t_{2}}{\sqrt{\hat{t}\left(1-\hat{t}\right)*\left(\frac{1}{n_{1}}+\;\frac{1}{n_{2}}\right)}}, \end{array}$$

where

(3b) $$\begin{array}{l} \hat{t}\;=\;\frac{T_{1}+\;T_{2}\;}{n_{1}+\;n_{2}} \end{array}$$

The summation of monthly population, mortality and morbidity counts, and the calculation of percentages in the EKS Benchmark data were calculated using R (RStudio version 1.1.383) statistical software (14). The Equation (5) standard deviations and Equation (6) Z statistics were calculated using Fortran code, while the R cor.test and lm functions were used to calculate Pearson correlations and linear regressions. Significance of correlations and Z statistics were defined at a 95% confidence level (*p* = 0.05) or better.

## Results

### Variation in Cattle Population, Mortality, and Morbidity

Monthly total population counts are shown in Figure 1B, while Figures 1C,D show total mortality (T_a_) and morbidity (B_a_) counts for all causes. From 2015 to 2018, total estimated head on feed increased from 1,579 in January 2015 to a maximum of 17,156 in December 2017, then decreased to a minimum of 1,078 in December 2018. During the 48-month period, the T_a_ and B_a_ counts varied in a manner roughly proportional to total population. Mortality counts rose from 2 in March 2015 to a peak of 188 in December 2017, then decreased to 4 in December 2018. After relatively constant morbidity counts under 200 during most of 2016, B_a_ counts increased to 807 in December 2016, decreased during the spring and summer of 2017, then rose to 995 in November 2017.

**Figure 2:**
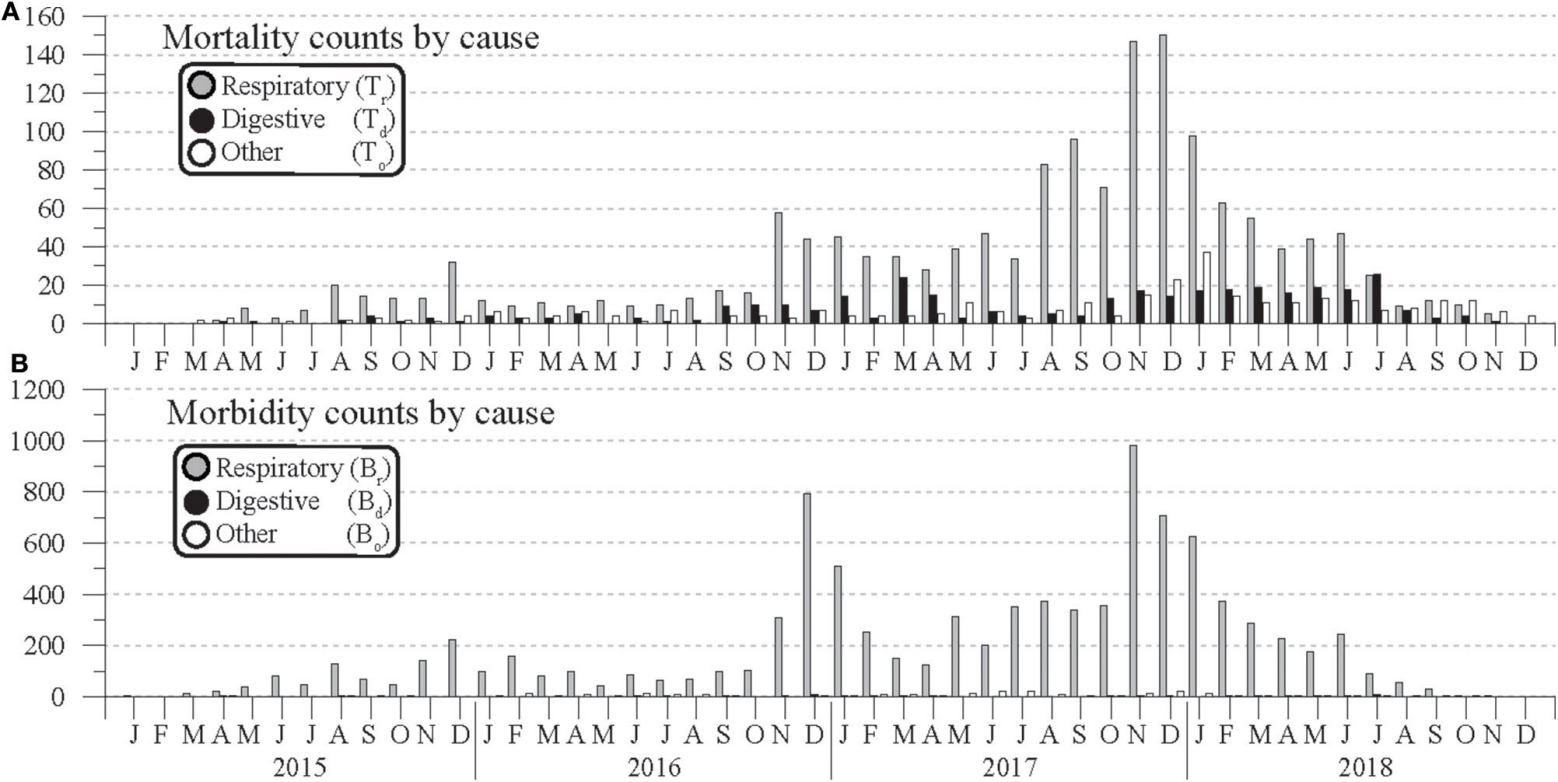
Monthly counts of **(A)** mortality and **(B)** morbidity by cause for cattle across 19 feedlots within a 96.6-km radius of Hereford, TX, in the Texas Panhandle from January 2015 to December 2018.

**Figure 3:**
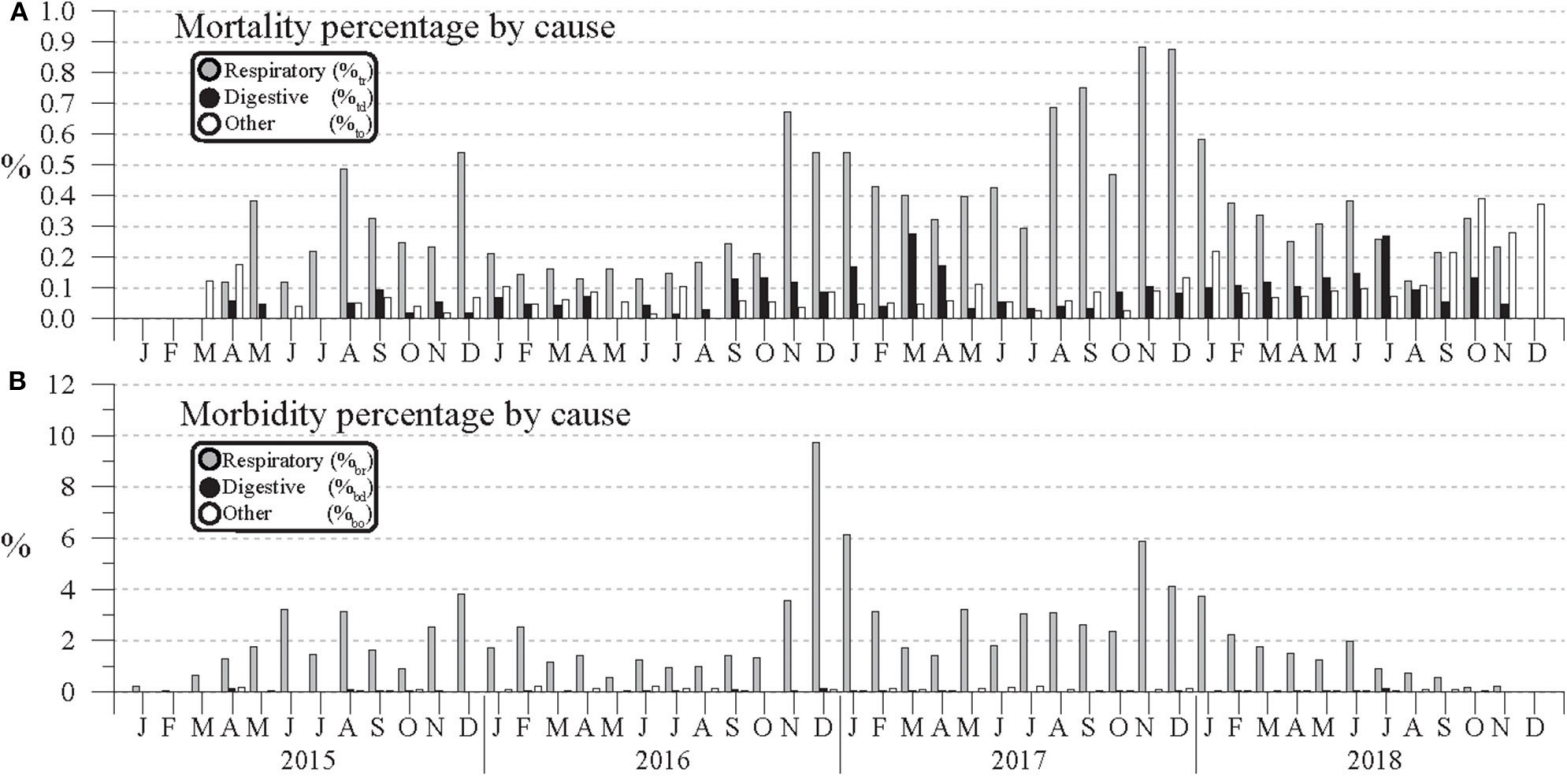
Monthly **(A)** mortality and **(B)** morbidity percentage by cause for cattle across 19 feedlots within a 96.6-km radius of Hereford, TX, in the Texas Panhandle from January 2015 to December 2018.

Mortality and morbidity counts by cause are plotted in Figures 2A,B. For both cases, respiratory causes were the most commonly reported, most noticeably in morbidity counts. Because these counts might be expected to track total head count, the corresponding Equations (1a,b) morbidity and mortality percentages were calculated and are plotted in Figures 3A,B. This normalization shows that respiratory mortality and morbidity counts are not strictly proportional to total head count. For example, B_r_ counts in Figure 2B peak in Nov. 2017, but Figure 3B's respiratory morbidity percentages peak in December 2016. Also, in Figure 2A the November 2017 respiratory mortality counts are more than double those of November 2016, but the Figure 3A respiratory percentages for those months are more comparable. During 2015–2019, monthly respiratory mortality percentages (%_tr_) peaked in the winter months, with the three highest values occurring in November 2016 (0.67 %), November 2017 (0.88 %), and December 2017 (0.87 %). Similarly, the three highest monthly morbidity percentages occurred in December 2016 (9.7 %), January 2017 (6.1 %), and November 2017 (5.9 %) from respiratory causes. By contrast, no clear seasonal variation in morbidity or mortality due to digestive or other causes seems apparent.

**Figure 4:**
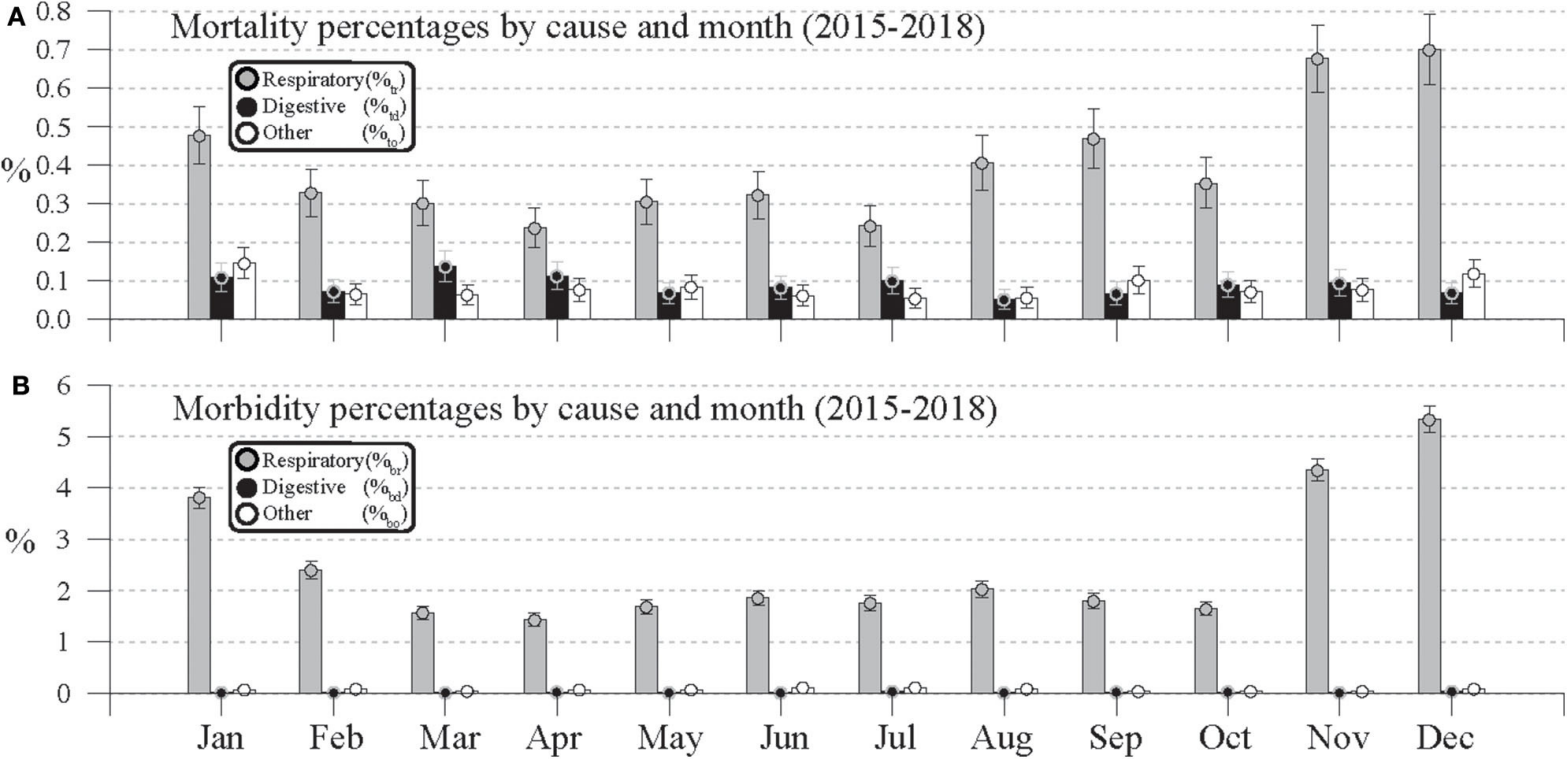
Monthly **(A)** mortality percentage and **(B)** morbidity percentages for respiratory, digestive, and other causes calculated from mortality, morbidity, and total head counts aggregated by month during January 2015 to December 2018.

When the Equation (1) mortality, morbidity, and population counts for each cause are aggregated by month across all 4 years, the resulting annual cycles of %_t_ and %_b_ in Figures 4A,B show significantly increased percentages for respiratory causes during the winter months. The ±1.96^*^σ uncertainty bars of the Figure 4A respiratory mortality percentages (%_tr_) for each month suggest significantly elevated death rates in November and December relative to October and January. Although the November and December t_r_ ratios are statistically indistinct (*Z* = 0.372, *p* = 0.710), the Z statistic for the difference in November and October ratios is 5.686 (*p* < 0.0001) and the statistic for the December and January ratios is 3.709 (*p* < 0.001). Although the January t_r_ ratio is significantly lower than December's, the January 95% uncertainty range for %_tr_ does not overlap with the intervals of February through July. The Z statistics comparing the January t_r_ ratio with the ratios of those months are all significantly different at 99% confidence interval (*p* < 0.01), which suggests that January might be included in a winter respiratory mortality season.

In Figure 4A, a secondary peak in respiratory mortality rate is also seen in August and September, whose t_r_ ratios are not significantly different at a 95% confidence level (*p* = 0.237). However, the August t_r_ ratio is significantly different from the July ratio (*p* < 0.001), and the September ratio is significantly different from October's (*p* < 0.05). Although Figure 4A shows evidence of seasonality in respiratory mortality, the 1.96^*^σ uncertainty bars for digestive and other causes consistently overlap, which indicates the lack of significant seasonal variation. Those uncertainty intervals also fall clearly below those of the same month's %_tr_ intervals, which shows that overall respiratory mortality was significantly greater than that from digestive or other causes during 2015–2018.

In Figure 4B's annual cycles of morbidity percentages, the values associated with respiratory causes (%_br_) clearly dominate those from digestive or other causes. Similar to Figure 4A's mortality percentages, the highest %_br_ values occur in November (4.34 %), December (5.34%), and January (3.81 %). However, given the small 1.96^*^σ intervals relative to those values, all of those percentages are significantly distinct from one another. Unlike the respiratory mortality percentages in Figure 4A, there is no evidence of a significant increase in respiratory morbidity during August and September.

### Correlation of Monthly Respiratory Mortality and Morbidity Rates vs. Monthly Temperature Conditions

**Figure 5:**
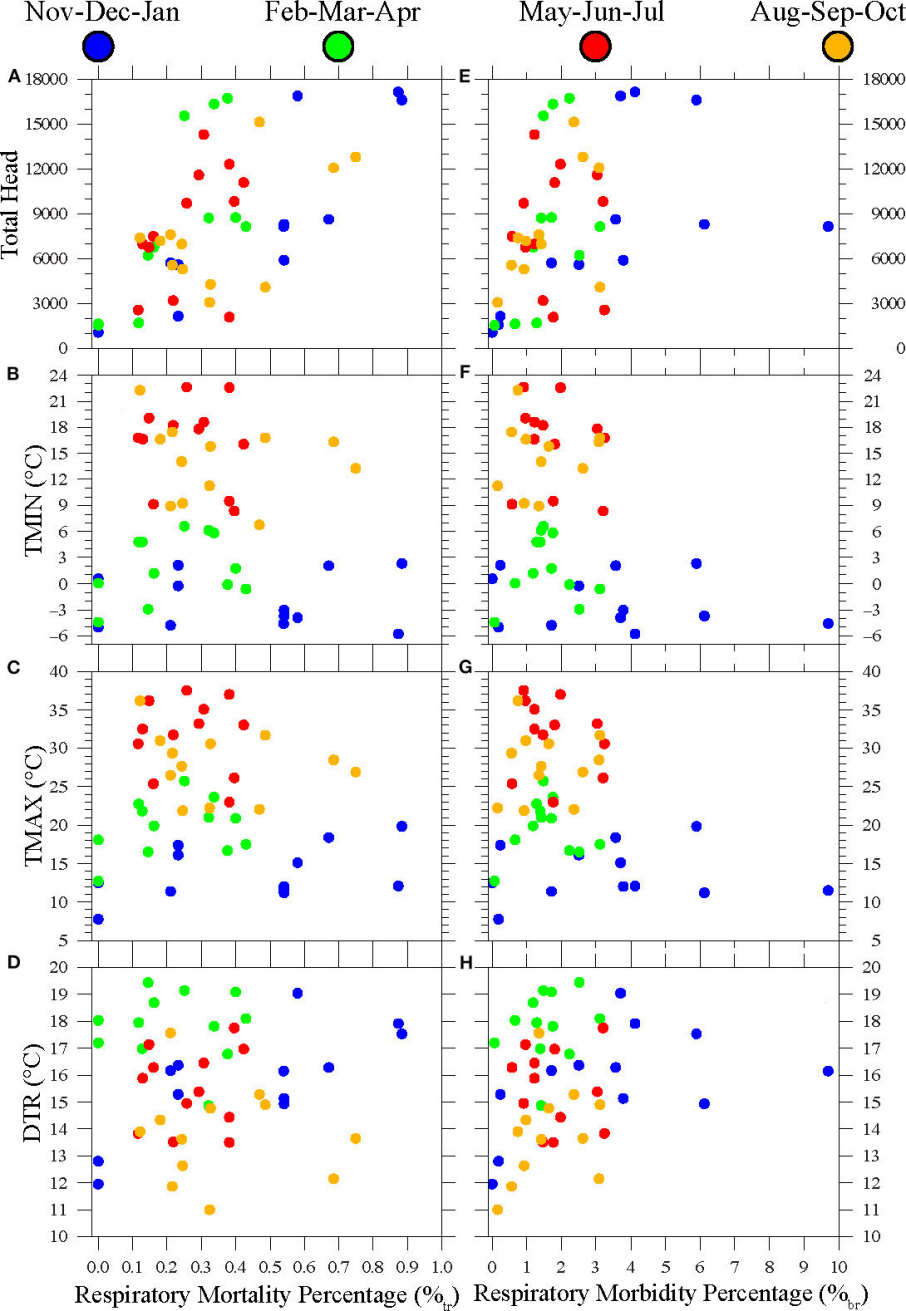
Scatterplots of **(A)** monthly respiratory mortality percentage (%tr) vs. monthly total head, **(B)** %tr vs. average monthly minimum temperatures (TMIN), **(C)** %tr vs. average monthly maximum temperatures (TMAX), **(D)** %tr vs. average monthly daily temperature range (DTR), **(E)** %tr vs. monthly total head, **(F)** monthly respiratory morbidity percentage (%br) vs. average monthly minimum temperatures (TMIN), **(G)** %br vs. average monthly maximum temperatures (TMAX), and **(H)** %br vs. average monthly maximum daily temperature range (DTR). All monthly head counts, %tr, and %br values were calculated across 19 feedyards within a 96.6-km radius of Hereford, Texas, during January 2015 to December 2018. All monthly temperature values were calculated from daily data from the Hereford Mesonet station.

In Figure 5, relationships between monthly respiratory mortality (%_tr_) and morbidity (%_br_) percentages and monthly population and temperature conditions during 2015–2018 are explored via scatterplots. The monthly mean minimum temperature (TMIN), maximum temperature (TMAX), and daily temperature range (DTR) values for Hereford TX during 2005–2018 can be found in Table 1 and are representative of the region's cold semi-arid climate conditions. During 2015–2018, Hereford TMIN varied between −5.8°C (December 2017) to 22.6°C (July 2018) while TMAX varied between 7.8°C (January 2015) to 37.6°C (July 2018). Monthly average DTR varied between 11.0°C (October 2018) and 19.4°C (February 2016). As noted in section Feed yard and Meteorological Data, Hereford monthly temperature conditions are considered to be representative of the temperature conditions of the feedyards considered here, which lie within a 96.6-km radius of the Hereford WTM site.

**Table 1:** Monthly average minimum temperature (Tmin), maximum temperature (Tmax), and daily temperature range (DTR) in degrees centigrade (°C) for Hereford Texas during 2005–2018.

	**Jan**	**Feb**	**Mar**	**Apr**	**May**	**Jun**	**Jul**	**Aug**	**Sep**	**Oct**	**Nov**	**Dec**
Tmax	11.5	13.5	18.8	22.5	27.0	23.8	32.7	32.1	28.3	22.8	17.3	10.9
Tmin	−5.5	−3.7	0.8	4.7	10.5	16.9	18.3	17.6	13.8	6.8	−0.2	−4.5
DTR	17.0	17.2	18.0	17.8	16.5	15.9	14.4	14.5	14.5	16.0	17.5	15.4

Figures 5A–D plot monthly %_tr_ for each month vs. monthly total head (a), TMIN (b), TMAX (c), and DTR (d). Figures 5E–H are similar scatterplots comparing monthly %_br_ vs. the same sequence of population and monthly mean temperature variables. Given the increased mortality and morbidity in Figures 4A,B during November, January, and February, the Figure 5 scatterpoints are colored to distinguish winter (November–December–January), spring (February–March–April), summer (May–June–July), and fall (August–September–October) months in both plot sequences.

Figure 5A shows a roughly linear scatter between monthly respiratory mortality percentages and population, with the 12 winter scatterpoints suggesting a semi-linear relationship. By contrast, the relatively random scatter of mortality percentages with TMIN and TMAX shows no obvious overall linear or nonlinear relationship (Figures 5B,C). However, those plots show that mortality percentages above 0.5% occurred in the winter or fall months. The two fall months with greater percentages occurred in August and September of 2017. Similarly, no clear relationship between DTR and mortality percentages over all seasons was seen in Figure 5D, but there was a tendency for winter mortality percentages to increase as mean monthly DTR increased during the winter months.

To test the significance of the Figures 5A–D scatter patterns, Pearson correlation values were calculated between monthly %_tr_ and concurrent TMIN, TMAX, DTR, and total head values (Table 2). The correlations showing significance at a 95% or better confidence level (*p* < 0.05) are generally consistent with the scatter patterns noted above. Correlations of %_tr_ percentages with population were significant over all 48 monthly data points (*r* = 0.637; *p* < 0.001) and for all seasons except summer. The correlation between winter mortality and population is Table 2's highest correlation (*r* = 0.877) and is significant at a 99.9% confidence level (*p* < 0.001). Apart from the correlations with population, the only other significant correlation was between %_tr_ and DTR during the winter months (*r* = 0.755; *p* < 0.01).

**Table 2:** Pearson correlations between temperature data, total head, and respiratory mortality rate from cattle at 19 feedlots in the Texas Panhandle from January 2015 to December 2018.

**Time period**	**DF^**e**^**	**Tmin^**f**^**	**Tmax^**g**^**	**DTR^**h**^**	**Total head**
Annual (Jan–Dec)	46	−0.121	0.115	0.073	0.637^***^
Winter^a^	10	0.003	0.428	0.755^**^	0.877^***^
Spring^b^	10	0.296	0.277	−0.072	0.721^**^
Summer^c^	10	−0.138	−0.102	0.124	0.454
Fall^d^	10	−0.162	−0.217	−0.122	0.581^*^

a *Winter: November–December–January*.

b *Spring: February–March–April*.

c *Summer: May–June–July*.

d *Fall: August–September–October*.

e *DF: degrees of freedom*.

f *Tmin: minimum temperature*.

g *Tmax: maximum temperature*.

h *DTR: daily temperature range*.

*** *P < 0.001*.

** *P < 0.01*.

* *P < 0.05*.

Table 2's significant winter DTR vs. total head correlations suggest that winter respiratory mortality rates might be better explained through the combined influence of those two factors. However, a linear regression that estimates winter %_tr_ based on both of those variables explains the same amount of variance (*r*^2^ = 0.769) that total head count alone does. Thus, the combined regression effect of head count and DTR is not additive. A regression based on DTR alone explains less %_tr_ variance (*r*^2^ = 0.569) than head count. Also, it is possible that the significant correlation between winter DTR and %_tr_ is due to chance. In addition to being significantly correlated with %_tr_, winter DTR is even more highly correlated with winter head count (*r* = 0.864, *p* < 0.001). Total head count vs. DTR correlations during the remaining spring (*r* = 0.022, *p* = 0.947), summer (*r* = 0.560, *p* = 0.058), and fall (*r* = 0.205, *p* = 0.522) seasons are all insignificant, which might be expected between total head count and a weather variable that should have no influence on feedyard stocking rates. Thus, Table 2's significant winter DTR vs. %_tr_ correlation may be due to a chance coincidence between increasing monthly winter total head counts and increasing winter DTR during 2015–2018 and is a possible example of type I error.

The Figures 5E–H scatterplots compare monthly %_br_ with concurrent total head count, TMIN, TMAX, and DTR show similar but weaker relationships than their %_tr_ counterparts in Figures 5A–D. The corresponding Pearson correlations and their statistical significance are found in Table 3. The Figure 5E scatter shows a significant relationship (*r* = 0.377, *p* < 0.01) for %_br_ to increase with total head over all seasons, but a stronger correlation was found between %_tr_ and total head in Table 2. Aside from a low but significant negative correlation (*r* = −0.312, *p* = 0.03) between %_br_ and TMIN over all seasons, there were no significant effects of varying TMIN or TMAX on %_br_ during any season. However, in Figures 5F,G, percentages above 4.0% are limited to the winter months. Monthly %_br_ showed no clear or significant relationship with DTR (Figure 5H). Although the Table 3 correlations between %_br_ and total head during the winter (*r* = 0.548, *p* = 0.065), spring (*r* = 0.475, *p* = 0.118), and fall months (*r* = 0.554, *p* = 0.062) are higher than the all-month correlation, they are not significant at a 95% confidence level.

**Table 3:** Correlations between temperature data, total head, and respiratory morbidity rate from cattle at 19 feedlots in the Texas Panhandle from January 2015 to December 2018.

**Time period**	**DF**	**Tmin^**e**^**	**Tmax^**f**^**	**DTR^**g**^**	**Total head**
Annual (Jan–Dec)	46	−0.312^*^	−0.284	0.205	0.377^**^
Winter^a^	10	−0.228	0.066	0.473	0.548
Spring^b^	10	0.003	0.074	0.203	0.475
Summer^c^	10	−0.174	−0.209	−0.096	−0.028
Fall^d^	10	−0.052	0.068	0.294	0.554

a *Winter: November, December, January*.

b *Spring: February, March, April*.

c *Summer: May, June, July*.

d *Fall: August, September, October*.

e *Tmin: minimum temperature*.

f *Tmax: maximum temperature*.

g *DTR: daily temperature range*.

** *P < 0.01*.

* *P < 0.05*.

## Discussion

Feedyard cattle morbidity with respect to respiratory disease peaked at 9.7% in the current study, whereas data from NAHMS (15) reported respiratory disease affecting 16.2% of all cattle in the United States. Data from smaller feedyards (1,000 to 8,000 head capacity) more closely resembles data from this study suggesting only 9.0% of cattle from those locations were affected by respiratory disease. Data from the NAHMS study also suggests that placement of cattle >317.5 kg reduced the percentage of cattle affected by respiratory illness from 21.2 to 8.8% compared to their smaller counterparts entering the feedyard at a weight <317.5 kg. Reported mortalities by month peaked at 0.88% in the data utilized for this study, whereas Loneragan et al. (16) reported an average mortality of 0.0126% utilizing data from 1994 to 1999. A 1978–1979 study in Canada also reported a mortality percentage of 0.134% across all types of cattle from 81 farmers (17). The fact that the greatest amount of morbidity and mortality were associated with respiratory disease was not a surprise based on previously reported data (15). The variation between data in these studies may be partially explained when considering the referenced studies are of national scope in comparison to a small geographical area of the Texas Panhandle. Regional management practices and breed type may vary greatly and partially explain the observed differences in health occurrence percentages.

Overall, morbidity and mortality were greatest in winter months. In addition to a tendency for increased winter mortality and morbidity, the Figure 3A respiratory mortality percentages, and to a lesser extent the respiratory morbidity percentages of Figure 3B, appear generally proportional to the Figure 1C monthly population counts. Stated otherwise, respiratory death and morbidity percentages were found to increase as the population of the feedyards increased. By contrast, similar variation was not evident in the digestive and other death and morbidity percentages. Monthly mortality percentages classified as digestive or other causes remained relatively stable across the 4 years, with the exception of elevated percentages of digestive-related deaths in March 2017 and July 2018, and increased percentages of deaths classified as other from October to December 2018 (Figure 3A). Although respiratory death and morbidity counts might be expected to track total population counts, their percentages should also be expected to be approximately constant unless there is a link between respiratory problems and population. However, here a proportionality seems evident between population and morbidity and mortality percentages. This is intuitively reasonable: as yards and lots become more densely populated, percentages of respiratory transmission, morbidity, and mortality might be expected to increase due to a possible increase in the probability of exposure. Data from a retrospective mortality study also reported respiratory disorders as the primary cause of death, accounting for greater than half of all mortality cases (16) followed by digestive causes of mortality.

The lack of seasonal patterns associated with digestive morbidity and mortality was surprising. Anecdotal evidence suggests that cold weather may alter feed delivery and intake patterns, and the changes in temperatures in the spring and fall may result in overeating and acidotic conditions. Another point of interest was the lack of a correlation between heat stress and morbidity and mortality. Vogel and Parrott (18) reported increased mortality from digestive issues from June to November of 0.18 to 0.47%, and similar to this study, digestive mortality was greatest in November and December. Conversely Elam (19) reported the greatest incidence of digestive issues the feedlot occurring during warmer weather possibly associated with a change in eating behavior. Concurrently, Mader et al. (20) and Hahn and Mader (21) also described alterations in feeding behavior, growth performance, and mortality percentages during periods of increased temperatures.

Anecdotal observations of health issues during extreme temperature swings were partially confirmed in this study as a link was observed between winter DTR and respiratory mortality percentage. This correlation is further confirmed as Cusack et al. (11) similarly asserted daily weather fluctuations and the incidence of BRD. However, these fluctuations do not correspond to temperature fluctuations in other seasons, most notably being absent in spring months. A study conducted by Cusack et al. (9) reported associations between BRD morbidity and mortality and weather in Australian feedlot cattle. Specifically, the authors found an association between minimum daily temperature and BRD morbidity. However, the aforementioned study focused on one feedlot over a two-month period, which contrasts with the current study which analyzed data on 19 feedyards across 4 years. As shown previously, however, DTR and stocking density are closely correlated in winter months over the 4-year period. Given the high correlation of these causally unrelated variables, making an association between winter DTR and morbidity based on our data may be an example of TYPE I error.

Trends from this analysis indicate that as the total head on feed increases, the percentages of respiratory-related morbidity increased (i.e., the greater the stocking density, the greater the risk of illness). Data from NAHMS (15) reported 9% of respiratory disease in cattle located in feedlot housing between 1,000 and 8,000 head; however, the percentages increased to 17.2% of cattle affected in feedlot housing >8,000 head. Yet, due to the possible design differences between the sampled feedyards, it may not fully explain the increased morbidity percentage. Increased contact between animals due to proximity or increased numbers of animals sharing a space such as a feed bunk or processing area may influence the incidence of disease (22). Additionally, commingling of cattle from different sources, often referred to as high-risk cattle, may increase the probability of a respiratory disease occurrence; however, the authors were unable to distinguish calf source from the database utilized for this analysis. This manuscript does not address many potential confounding variables including, but not limited to, gender, breed type, age, misdiagnosis, management practices, etc., but attempts to objectively describe the data from a high vantage point evaluating the relationships of ambient temperature, morbidity, and mortality. Undoubtedly, these data will warrant future analysis to further stratify these data to recognize specific patterns that producers may be able to use to predict feedlot morbidity and mortality in relationship to seasonal weather patterns.

## Conclusions

Overall, there is no strong correlation between ambient temperature and cattle health. However, both morbidity and mortality percentages increase during winter months, with the majority of the causality associated with respiratory issues. Additionally, increases in populations of cattle within a feedlot resulted in increased percentage of health events. There was no correlation of health patterns with summer temperature patterns. Collectively, these data report correlations that both coincide and contradict with dogmas surrounding climate and health. Further exploration across different regions with the incorporation of more cattle and feedlot demographic data may be warranted to further elucidate the relationship between ambient temperature patterns and feedlot cattle health and well-being.

## Data Availability Statement

The datasets generated for this study are available on request to the corresponding author.

## Ethics Statement

Ethical approval was not required according to national/local legislation because the described study did not use live animals. Rather, this manuscript describes a retrospective analysis of cattle data acquired from a database of U.S. feedyard data.

## Author Contributions

PB, JC, and SM initiated the initial study design. PB and SM collected the data. Data was analyzed by SM. The manuscript was written by PB, NB, and SM. All authors reviewed drafts of the manuscript and provided approval prior to submission.

## Conflict of Interest

The authors declare that the research was conducted in the absence of any commercial or financial relationships that could be construed as a potential conflict of interest.

## Abbreviations

BRD: bovine respiratory disease
DTR: daily temperature range
TMIN: minimum temperature
TMAX: maximum temperature
EKS: Elanco Knowledge Solutions.
